# A set of genes conserved in sequence and expression traces back the establishment of multicellularity in social amoebae

**DOI:** 10.1186/s12864-016-3223-z

**Published:** 2016-11-04

**Authors:** Christina Schilde, Hajara M. Lawal, Angelika A. Noegel, Ludwig Eichinger, Pauline Schaap, Gernot Glöckner

**Affiliations:** 1 Division of Cell and Developmental Biology, School of Life Sciences, University of Dundee, DD15EH Dundee, UK; 2 Institute for Biochemistry I, Medical Faculty, University of Cologne, Cologne, Germany; 3 Institute for Freshwater Ecology and Inland Fisheries, IGB, Berlin, Germany

**Keywords:** Developmental program, Evolution, Dictyostelia, Expression pattern conservation, Multicellularity, Developmental genes

## Abstract

**Background:**

The developmental cycle of Dictyostelid amoebae represents an early form of multicellularity with cell type differentiation. Mutant studies in the model *Dictyostelium discoideum* revealed that its developmental program integrates the actions of genes involved in signal transduction, adhesion, motility, autophagy and cell wall and matrix biosynthesis. However, due to functional redundancy and fail safe options not required in the laboratory, this single organism approach cannot capture all essential genes.

To understand how multicellular organisms evolved, it is essential to recognize both the conserved core features of their developmental programs and the gene modifications that instigated phenotypic innovation. For complex organisms, such as animals, this is not within easy reach, but it is feasible for less complex forms, such as the Dictyostelid social amoebas.

**Results:**

We compared global profiles of gene expression during the development of four social amoebae species that represent 600 mya of Dictyostelia evolution, and identified orthologous conserved genes with similar developmental up-regulation of expression using three different methods. For validation, we disrupted five genes of this core set and examined the phenotypic consequences.

**Conclusion:**

At least 71 of the developmentally regulated genes that were identified with all methods were likely to be already present in the last ancestor of all Dictyostelia. The lack of phenotypic changes in null mutants indicates that even highly conserved genes either participate in functionally redundant pathways or are necessary for developmental progression under adverse, non-standard laboratory conditions. Both mechanisms provide robustness to the developmental program, but impose a limit on the information that can be obtained from deleting single genes.

**Electronic supplementary material:**

The online version of this article (doi:10.1186/s12864-016-3223-z) contains supplementary material, which is available to authorized users.

## Background

The information encoded in a genome mirrors the potential of an organism to manifest a corporeal form that can adapt to a changing environment. To achieve this flexibility, a set of “housekeeping” genes, which define the shape and basic physiology of the organism, is expressed constitutively, while other genes are only expressed when needed. Regulation of gene expression is therefore a major mechanism to enable organisms to respond for example to environmental changes [1]. Such regulatory events can be fairly straightforward, as in prokaryote responses to nutrient availability, where the nutrient enters the cells and acts on a transcriptional regulator [2]. However, in general, the processes leading from signal detection to gene expression are more complex and this is particularly the case in multicellular organisms.

During development of multicellular organisms, a range of cellular functions such as cell differentiation, cell division and cell movement have to be coordinated by intercellular communication to generate a functional final form. The regulatory circuitry to achieve this feat, and the genes encoding its components, cannot have all appeared at the same time. Rather, pre-existing genes have been co-opted for novel roles. Progressive re-iteration of gene co-option, combined with evolution of novel coding sequences by mutation and occasional horizontal gene transfer, may gradually have generated the complex regulatory mechanisms that control the development of modern multicellular organisms [3]. Changes in the cis-regulatory regions of genes, allowing genes to be expressed at novel stages or locations in the developing form, appeared to have played a crucial role in generating morphological diversity in animals and plants, but alteration of gene function after gene duplication or by acquisition of novel functional domains will also have contributed to the emergence of developmental complexity.

**Fig. 1:**
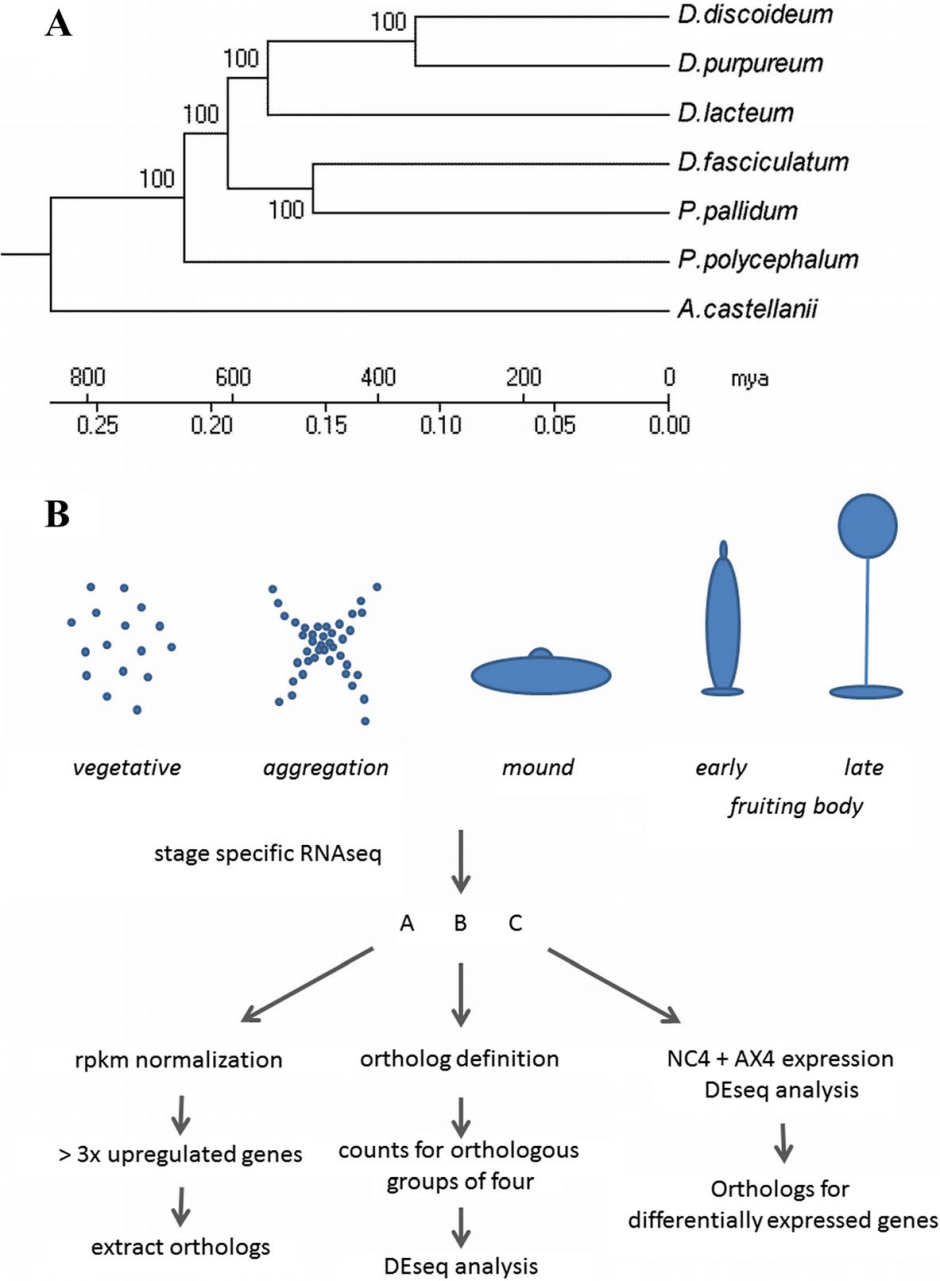
**a** The phylogeny of Dictyostelida. A maximum likelihood phylogeny of Amoebozoa with sequenced genomes based on a concatenated data set of 30 genes. Orthologs between all species were selected as in [27]. The tree was rooted with metazoa, plant, and fungi orthologs (not shown). Scale bar is in millions of years (mya), adjusted using dated splits of animals and plants (520 and 670 mya, respectively). **b** The morphological stages of sampling and a flowgram for the three different analysis methods of the RNAseq data. Morphological stages are purely schematic similar to DD stages, the fruiting body morphology differs between species

To understand how multicellular organisms evolved, it is essential to recognize the core conserved features of their developmental program and the gene modifications that caused phenotypic innovation. For complex organisms, such as animals, this is a daunting task, which is complicated by the fact that the unicellular ancestor is long extinct or has meanwhile evolved along a different trajectory. However, it is feasible for less complex forms, such as the Dictyostelid social amoebas, which have a conditional form of multicellularity. Dictyostelia initially feed as unicellular amoebas on bacteria and enter multicellular development by aggregation, when starved. The aggregates transform into migrating slug-shaped structures and finally into fruiting bodies that consist of a spore mass and up to four different cell types to carry the spore mass aloft. In the model organism *D.discoideum* (DD), several signal molecules and direct cell-cell interactions that coordinate morphogenesis and trigger cell-type specialization, and many components of the pathways that process these stimuli have been identified [3, 4]. Even the correct positioning of nucleosomes seems to be influenced by the ability of DD to form multicellular structures [5]. A molecular phylogeny based on rRNA and nuclear encoded protein sequences subdivides all known Dictyostelia into two main branches each containing two major and some minor groupings, with DD residing in group 4 [6–8] (Fig. 1). The split between the two main branches from the last common ancestor was dated at around 600 million years ago, indicating that this form of multicellularity emerged almost as long ago as that of the animal kingdom with around 700 million years [9]. Phenotypic analysis revealed that groups 1–3 contain species that predominantly form small clustered or branched fruiting structures with maximally two cell types. Many species in these groups have retained encystation, the unicellular life cycle of their amoebozoan ancestors as an additional survival strategy. Group 4 species form larger solitary and unbranched fruiting bodies with up to five cell types and have lost encystation entirely [10].

Microarray and RNAseq based transcriptomics in DD revealed that at least 25 % of the DD genes are affected by the transition from growth to multicellular development [11, 12]. While members of the basic cellular machinery, such as genes encoding ribosomal proteins, are down-regulated, more than 2000 genes are up-regulated. These genes are likely to be involved in either regulating cell differentiation or in defining the differentiated state. However, some of these genes may be subject to co- or mis-regulation, e.g. hitchhiking effects from neighboring genes or suppression of global negative regulators. *D. purpureum*, another group 4 species, showed a similar profile, indicating broad conservation of the developmental gene expression in group 4 [13].

In this work we used comparative transcriptomics of species across the entire Dictyostelid phylogeny to define the core set of orthologous developmentally regulated genes. This approach provides the basis for the discovery of currently missing components or even entire pathways that control development and for subsequent studies investigating how evolutionary changes in the core set led to phenotypic innovation. For five core set genes we tested the phenotypic consequences of gene disruption.

## Methods

### Species and sample preparation

*D. discoideum* NC4 (DD), *Dictyostelium lacteum* (DL), *Polysphondylium pallidum* PN500 (PP) and *Dictyostelium fasciculatum* SH3 (DF) were grown in association with *E.coli* 281 in 10 mM phosphate buffer, pH 6.5 at 150 rpm and 21 °C, until a density of 2-3×10^6^ cells/ml was reached. Cells were washed free from bacteria and either frozen directly at −80 °C for the t = 0 h time point, or plated on phosphate buffered agar, which contained 0.5 % charcoal for DL, PP and DF to improve synchronous development. The progression of development was monitored and cells were harvested at four developmental stages defined by their morphology– early aggregation, mound, early and late fruiting bodies (Fig. 1b). Cells were harvested in chilled phosphate buffer and cell pellets were snap-frozen on dry ice and stored at −80 °C until RNA extraction.

### Sequencing and mapping

RNA was extracted using the Qiagen RNA easy kit with samples from culminating fruiting bodies being vortexed for 10 min with glass beads to break spore and stalk cell walls. The mRNAs were converted to a sequencing ready library with the mRNA kit from Illumina, and paired end sequenced using an Illumina HiSeq instrument. The TopHat [14] pipeline with the HTseq script [15] was used to count the number of reads mapped to specific genes in each genome. Individual mapping results were normalized to the total number of reads obtained for each sample and rpkm (reads per kilobase gene sequence per million) values were calculated for each gene.

### Criteria for definition of developmental expression

Earlier results suggested that growth-specific genes are down-regulated upon entering the developmental cycle, while the expression of many development-specific genes is turned on or up-regulated. Thus, for our analysis we considered only developmentally upregulated genes. We first assessed, which genes in each species have expression data (Table 1a). To define upregulated expression we used three different methods (Table 1b; Fig. 1b). Each method has its drawbacks and advantages and a combination thus provides more robust results. The union of the resulting candidate genes would capture all potential genes of interest (the core set of developmentally upregulated genes) while the intersection of all methods would provide the most robust set of potential developmentally important genes. Method A relies on normalized counts only. A threshold of at least 20 reads per gene was set to exclude weakly expressed genes. A threshold for upregulated expression of the normalized counts (reads per kilobase per million sequencing reads; rpkm) of at least three times between the vegetative growth state and any other time point was set. These relaxed criteria can capture genes of which the expression pattern differs slightly between species but the false positive rate with such a threshold approach can be high [16]. With method B we defined orthologs between all species and used the expression of these orthologous genes as replicates in a DEseq analysis. Here we used the NC4 data of DD only to be compatible with the data of the other species. With this method the false positive rate might be lowest, but we might fail to capture all relevant genes. The third method (C) relied on the definition of upregulated genes in DD by using our NC4 and AX4 data available from earlier results [13]. Axenic and xenic growth conditions and the slightly different genome background of the two strains might lead to a higher false negative detection rate. The resulting developmentally upregulated genes were then categorized as species-specific if no ortholog to other species could be detected. Only developmentally upregulated genes with at least one ortholog in one of the other species were further analysed. For methods B and C we used DEseq [17] to define significantly developmentally upregulated genes with a false positive detection rate of 10 %.

**Table 1:** Overview of differentially expressed genes and orthologs in social amoebae

A: orthologs and expression counts				
	DD	DL	DF	PP
Number of genes	12319	10232	11879	11440
Expressed in data set	11549	10216	11854	11315
Ortholog families (ORTHOMCL)	7290	7172	7316	7114
B: definition of expression sets with different methods				
	Orthologs between all species		5895	
				In developmental set
A	≥ 3x developmentally upregulated	DD DL DF PP	2352 2895 2605 2955	776
B	Differentially expressed orthologs^a^			150
C		all	DD specific	
	Differentially expressed genes DD^a^	493	250	243

^a^DEseq; 10 % false positive detection rate

### Definition of orthologous relationships

We employed Augustus [18] together with our RNAseq data to improve the initial gene prediction of DF, PP and DL. While the overall number of predicted genes remained nearly the same, the gene model prediction had improved considerably based on our manual analysis of randomly selected genes. For DD we relied on the manually curated database at http://dictybase.org [19]. The improved predicted gene set was translated to protein sequences and grouped into gene families using OrthoMCL [20]. We used the default values of OrthoMCL for this approach and compared our results to the web server of this program (http://www.orthomcl.org/orthomcl/).

### Enrichment analysis

The translated gene sequences were matched against a refseq database of proteins from NCBI (Version from October 2013). GO terms were determined using the interproscan pipeline (http://www.ebi.ac.uk/interpro/; [21]) and the online tool Generic GO term finder [22]. GO term enrichment was done using REVIGO [23].

### KO mutant analysis

#### Selection of putative sporulation/encystation genes

From the set of genes (Additional file 1: Table S1) that were conserved in DD, DL, PP and DF and were over 3-fold upregulated during development of at least three species, closest homologs were sought by BLASTp search in the *Acanthamoeba castellanii* and *Physarum polycephalum* genomes [24, 25] at *e*-values < 0.001. From this set, genes were selected that were both upregulated in spores over stalk cells in PP, as well as upregulated in PP encystation. From this subset of 25 genes, five genes were selected for gene disruption (Additional file 2: Table S3).

#### Gene disruption

We used the AX2 strain, which is the axenic version of the original NC4 isolated strain, for these experiments. Details of strain histories can be obtained from the dictybase.org web page. For gene disruption two fragments (KO1 and KO2) of ~ 1 kb for each gene were amplified using the primer pairs listed in Additional file 2: Table S3. The fragments were digested with KpnI/HindIII or BamHI/NotI, using restriction sites incorporated in primer design, and sequentially inserted into KpnI/HindIII and BamHI/NotI digested plasmid pLPBLP [26] to flank the LoxP-Bsr selection cassette. AX2 cells were transformed with the KpnI/NotI insert that was excised from the plasmid, together with 1 μg of the KO1-5’KpnI and KO2-3’NotI primers to assist homologous recombination. Transformants were selected at 5 μg/ml blasticidinS (Invivogen). Genomic DNA from blasticidin resistant clones was extracted and screened by three PCR reactions (Additional file 3: Figure S4) to diagnose target gene disruption. Several knock-out (KO) clones and clones carrying random insert integrations (RI) were identified for each gene. For test of multicellular development, cells were harvested and plated on non-nutrient agar at 10^6^ cells/cm^2^ and 21 °C.

## Results

### Genome sequences and improved gene prediction using transcript data

The genomes of representative species of group 1 (DF), group 2 (PP) and group 4 (DD) were sequenced previously [27] and for complete or nearly complete representation of the genetic depth of Dictyostelia, we also sequenced the genome of the group 3 species DL [28]. With a size of 23 Mb, this is the smallest genome completed thus far, compared to the 31–35 Mb genomes of the other Dictyostelia. The DL genome has nevertheless about the same number of genes, indicating constraints for gene loss and retention in Dictyostelia.

The original gene model prediction of DF and PP was based on training of geneid [29] with a limited set of transcript data from 454 sequencing runs. In the course of manual curation, we detected problems in about 25 % of the gene models with respect to intron positions, overly long introns and inappropriately fused or split genes. To improve gene prediction for subsequent analyses we incorporated all available RNAseq data, including the RNAseq data that is used for expression profiling in this work. We trained the more advanced gene prediction program Augustus [30] with cDNA sequences and repeated the genome analysis. This yielded comparable numbers of predicted genes in DF, PP and DL, with gene numbers in DF and PP being similar to the previous prediction. Manual inspection of gene models confirmed the overall superiority of this new prediction over the previous one. No corrections were made to the DD genome, which was already manually curated [19]. The new gene model data are available via the Social Amoebae Comparative Genome Browser (SACGB) database [31].

### Defining orthologs across the four genomes

A prerequisite for species comparisons is an understanding of the evolutionary relationships between genes. Since genomes generally go through expansion, shrinkage and loss of gene families, orthologous genes, particularly within large gene families, cannot always be assigned. To avoid incorrect assignments, we did not attempt to group single genes, but instead defined gene families. Using the ORTHOMCL algorithm [20], we defined 8903 gene families consisting of at least two members, irrespective of their species affiliation. This number also includes genes that only have paralogs in the same species (i.e. species-specific gene duplications) without similar genes in another species. The largest family consisted of 208 genes (52 DD; 80 DL; 60 PP; 16 DF). 5016 families had exactly 1 member per species and all orthologous groups present in all species sum up to 5763. Including families that only existed in a single taxon, we defined over 7000 orthologous groups for each species (Table 1a).

### Gene expression during multicellular development

Between species there are considerable differences in the time required to aggregate and form fruiting bodies. To be able to compare the transcriptional profiles of species from different taxon groups, we isolated RNAs when species had reached a specific developmental stage rather than a specific time point after starvation. The chosen stages were growth stage, early aggregation, mounds, early and late fruiting body formation. The species DL, PP and DF do not develop very synchronously, which means that stage-selected RNAs are to some extent intermixed with RNAs from earlier or later stages. RNA was sequenced using Illumina technology. The majority of genes in the genomes of all 4 species are also represented in their transcriptome (Table 1).

For method A the mapped reads were normalized to yield rpkm values (reads per kilobase per million) to allow comparison of expression levels at different stages within one species. The incomplete synchrony of development of DL, PP and DF compared to DD caused quenching of expression differences between developmental stages. We therefore decided to define genes as developmentally upregulated if the normalized expression (rpkm values) at any developmental stage was at least 3-fold higher than at the *t* = 0 h growth stage. By this definition from 2352 to 2955 genes are developmentally upregulated in the different species (Table 1), but nearly half of the upregulated genes in each species have no identifiable counterpart in the other genomes. In larger gene families no clear orthology relationships exists. In this case we reasoned that similar expression profiles might confer similar functions and grouped family members with similar expression patterns together. We also included genes, which have identifiable orthologs to DD in three species only and are developmentally expressed in all three. This resulted in a set of 776 orthologous genes (Additional file 1: Table S1, method A).

For method B we first defined orthology relationships between all genes of the four species (Table 1b). Then we extracted all groups with expression data and treated the data sets of DL, DF, and PP as biological replicates of DD. The subsequent DEseq analysis yielded 150 developmentally upregulated genes (Additional file 1: Table S1, method B).

For method C we employed the read counts obtained from our sequences and the freely available AX4 data [13] from the same time points. A principal component analysis using cummeRbund [14] shows that the largest difference in gene expression occurs between vegetative growth (t = 0 h) and all other time points (Additional file 3: Figure S1). This is in agreement with previous RNA profiling of DD development [11]. Of the 493 developmentally upregulated genes only 243 have a detectable ortholog in at least one of the other species (Table 1b). These are listed in Additional file 1: Table S1.

**Fig. 2:**
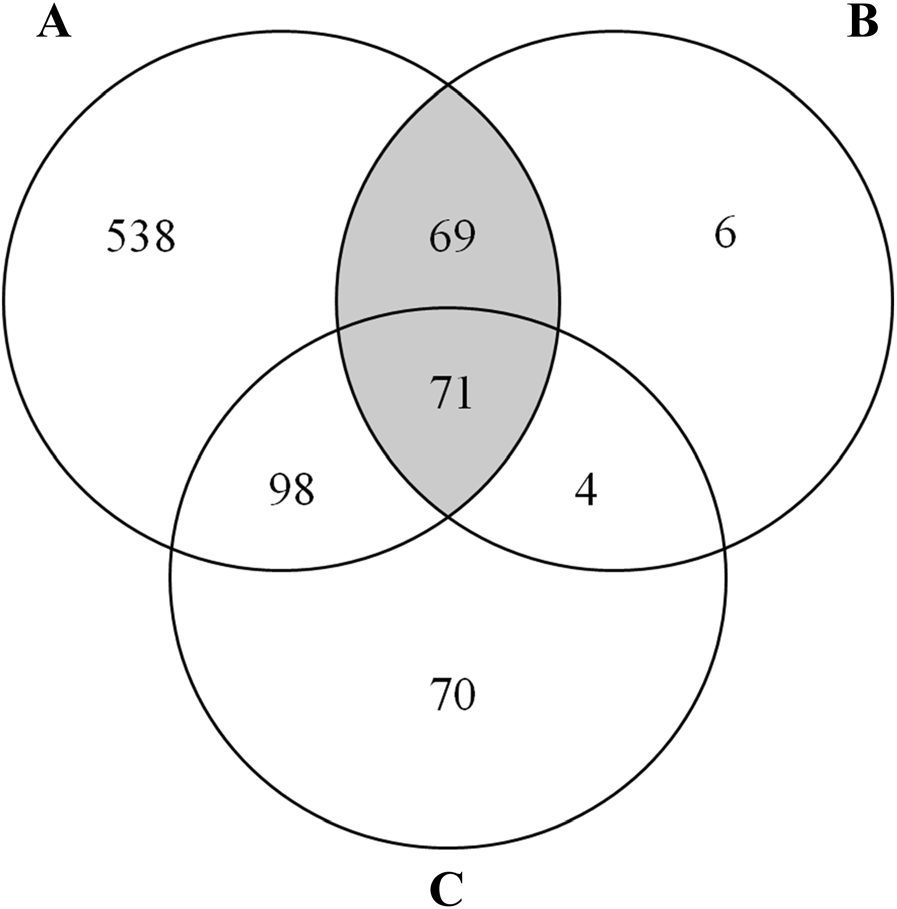
A Venn diagram showing the resulting numbers of genes from the three methods. **a**, **b**, and **c** are described in Table 1. The grey area highlights the intersection, where an enrichment of GO terms was observed (see Fig. 3)

The three methods yielded different numbers of potential conserved developmentally expressed genes. A comparison of the gene list revealed that 71 genes were detected with all methods (Fig. 2).

As expected method A yielded the highest number of genes and the overlap to the other gene sets is low indicating a high false positive discovery rate. Method B yields the lowest number of genes but nearly all of these detected genes were also observed with other methods indicating a low false positive discovery rate.

The expression peak time point of each gene in the defined set was analyzed in respect to its conservation (% identity) between DD and DF (Additional file 1: Table S1). A statistical analysis (Wilcoxon-Mann–Whitney-Test) showed that the conservation of genes with a peak expression at time point 16 h or 20 h (t_3 and t_4 in Additional file 3: Figure S3) is higher than at 4 h (t_1 in Additional file 3: Figure S3). This finding is in agreement with earlier results [32].

### GO term enrichment analysis

**Fig. 3:**
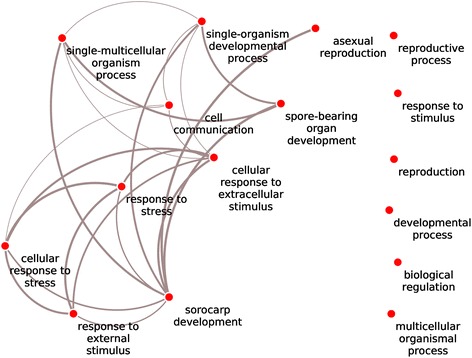
GO term enrichment in the 140 genes (*grey* area from Fig. 2). The generic GO term finder at http://go.princeton.edu/cgi-bin/GOTermFinder [22] was used to find significantly enriched GO terms in the core set of developmentally regulated genes. The complete protein set of all species was screened for GO terms using the interproscan algorithm [21]. The data were reformatted to the gaf file format and fed into the GoTermFinder program. To visualize the results REVIGO [23] was used. The network was analysed with Cytoscape (http://www.cytoscape.org/). Connections between enriched Go terms are shown as light grey lines

To assign functions to the core set genes, their protein functional domains were first analysed using interproscan [21], and the domains were next mapped to Gene Ontology (GO) categories. We tested for potential enrichment of GO terms using the GO term finder [22]. The complete set of defined developmentally expressed genes (859) was enriched, among others, in terms like signal transduction, regulation, cell communication (Additional file 3: Figure S2). We then analysed the genes in the different intersections (defined by two or three methods) separately. Only in intersection A|B and A|B|C we found Go term enrichments. Figure 3 shows a network analysis of the enriched terms of this intersection from methods A|B (140 genes; grey area in Fig. 2). The network connects stress responses to developmental processes and communication (Fig. 3).

### Comparison to characterized mutants

To date more than 1000 DD mutants targeting more than 600 genes have been described and most are available via the Dicty Stock Center (http://dictybase.org/StockCenter/StockCenter.html). 652 mutant strains, including null mutants, overexpressors, and multiple gene manipulations, showed a developmental defect. We found that 480 of the underlying affected genes have an ortholog in all the other three species. Thus, genes with phenotypic consequences upon manipulation are enriched in the orthologous set, since 73 % (480 of 652) of the genes defined by their impact on the development are also detectable in all other species but only 48 % of all DD genes (5895 of 12319).

Only 186 genes within the developmental mutant collection of DD appeared to be developmentally upregulated in DD (Additional file 4: Table S2). Of these 186 genes, 33 do not have a detectable ortholog in at least one of the other three species and 20 have no ortholog in all three species. Interestingly, among the latter 20 developmentally expressed DD genes with no detectable counterpart in all the other genomes are four coding for proteins related to spore formation and two involved in adhesion indicating species-specific evolution in these categories. The most prominent group of genes with orthologs in only a subset of species is again involved in adhesion (5 of 33). Fortyfour of the 186 DD genes are present as orthologs in all other species but appear not to be developmentally regulated in all species. The remaining 89 genes have orthologs and are also developmentally upregulated (according to method A).

### KO mutant analysis of selected genes

To assess the combined predictive value of deep conservation within Amoebozoa and conservation of gene regulation within Dictyostelia for an essential biological role of genes, we selected a set of orthologous genes that was developmentally upregulated in at least three out of four Dictyostelia and had likely orthologs in the solitary amoebozoa *Acanthamoeba castellanii* and PP. DD spore formation is evolutionary derived from encystation, sharing a core signalling pathway [33, 34]. From the above set, we selected five genes for knock-out by homologous recombination (Additional file 3: Figure S4). These genes, DDB_G0275521, DDB_G0287037 DDB_G0288963, DDB_G0269826 and DDB_G0272550 were both spore-enriched in PP and upregulated during encystation. DDB_G0272550 could not be knocked out despite three attempts. For none of the four obtained knock-outs, we noted any difference in developmental progression compared to wild-type. Fruiting bodies, stalks and spores looked normal and there were no significant differences in sporulation efficiency and spore viability (Additional file 3: Figure S5, S6A, B). Chimeric development in a 1:1 starter mix with wild-type cells over five cycles of growth and fruiting body formation also did not result in marked under- or over-representation of the knock-out clones in the spores (Additional file 3: Figure S6 C). From this small set, it therefore appears that predicting essential roles for genes from deep conservation and conserved developmental regulation remains challenging. However, the selected genes come from the set defined by method A only, and thus may have functions which are not essential for the developmental progression.

## Discussion

Developmental processes depend on sophisticated regulatory networks and specialized functions, which must be integrated with the normal cellular machinery. We set out to define a minimal set of genes recruited for developmental purposes, which were already present in the last common ancestor (LCA) of all social amoebae. We assumed that such genes would be up-regulated upon entering the cycle, while other genes that are essential for development but also required for growth, would not be affected. The developmentally up-regulated set may provide insights into the evolutionary mechanisms by which the developmental cycle was established. A comparison between two group 4 species, DD and *D. purpureum* showed high conservation of developmental gene expression [13]. It was however, unclear whether this conservation is restricted to group 4 or whether it also extends to the other major groups of social amoebae. Comparative genome analysis of *D. purpureum* and DD also showed that despite their considerable large evolutionary distance, gene synteny is still present [35]. Synteny was not observed between genomes outside this group, indicating low overall conservation [27], making the processes that are conserved all the more relevant.

Co-occurrence networks can be used to trace specific functions during evolution. Such networks tend to have a strong correlation between co-occurrence and co-expression [36]. Our study might be viewed as a first attempt to define co-occurrence networks for the developmental cycle.

### Methods to robustly define core developmental genes

Previous studies showed that at least 25 % of all DD genes alter their expression upon entering the developmental cycle [11, 13]. This was also the case for all four species examined by us. Using three methods with different strengths and weaknesses we defined a set of genes likely involved in social amoebae developmental processes since the LCA of all Dictyostelia emerged. With the methods B and C we wanted to define minimal gene sets by using strict statistical measures for differential expression. Replicate transcriptional profiles were previously generated for DD AX4 [13], which provided a biological replicate for our DD NC4 data. Method B stringently captures highly conserved genes and developmental programs by using species data as “evolutionary” replicates. Method A was designed to capture even genes with roles in development where transcriptional profiles differ slightly between species. We cannot, however find genes, where the orthology relationship between species has been masked during the long evolutionary separation of the species.

For some genes we may have not detected their developmental expression pattern in one or another species due to the increased complexity of growth and development on plates. Thus, method A includes orthologous genes, which are developmentally upregulated in three taxa only. Indeed, in this subset we found a gene, RegA, which was previously shown to be developmentally upregulated in the axenic AX4 strain of DD and found to be important for the phosphorelay system [37]. It showed no expression increase in the NC4 wild type strain of DD in our data set, but has developmentally upregulated counterparts in all other genomes. This finding emphasizes that the inclusion of such genes is justified.

Genes in the intersection A|B are strongly enriched in gene ontology terms related to developmental processes. However, of these 140 genes a large number is not yet functionally characterized (Additional file 1: Table S1) Furthermore, several of these genes have no detectable domain structure which would provide some hints for their function. Thus, our analysis opens up an alley to characterize developmental genes further. No GO term enrichment could be detected in genes defined with method A and C but not B (98 genes).

### Mutants in the developmental cycle

Not all genes that cause developmental changes after manipulation are developmentally up-regulated. Currently, 652 mutated genes with descriptions of developmental defects are listed in dictybase (http://dictybase.org).

We found, that 186 of these genes are developmentally upregulated in DD, but only half of these genes are also developmentally regulated in the other three investigated species or even present. Likewise, half of all developmentally regulated genes in each organism have no detectable developmentally regulated counterpart in the other species (data for DF, DL, PP not shown). These mutants mainly stem from screens for mutant phenotypes, and thus randomly pick up genes from our core set and species-specific developmental genes. Among the genes with described mutant phenotypes not detectable in more than 2 of the investigated species are genes overrepresented with functions in adhesion and spore formation. Adhesion gene variability enables kin recognition and exclusion of other species from fruiting body formation [38]. Spore differentiation on the other hand involves a number of coat proteins, where possibly the structure but not the sequence has constraints in evolvability. Both functions are presumably subject to species-specific modulation.

The occurrence of mutant phenotypes for genes of the non-core set indicates that despite lack of conservation, such genes still have indispensable roles in the developmental cycle. Some of these genes likely represent species-specific additions to the cycle, but others might have evolved beyond recognizable similarity.

Our attempts to investigate the core set further by constructing mutant strains of some conserved genes yielded no discernable phenotype for four genes and one gene that might be essential also in the vegetative state. Apparently, lack of consequences from the loss of a gene might lead to the conclusion that this gene is dispensable. However, high conservation of sequence and expression pattern over long evolutionary distances can only be maintained if it remains under purifying selection. Laboratory conditions are far more constant than natural environments and thus might require less sophisticated and robust gene sets for the developmental cycle. On the other hand, not all possible phenotypes are testable in the laboratory and thus might have escaped our notion [39]. The 89 described mutant phenotypes of genes of the core set could therefore represent the observable phenotypes and essentiality must be defined in respect to environmental and not laboratory conditions.

## Conclusion

Generation of specialized cells and tissues during a developmental cycle enabled higher order complexity of organisms. Due to the large number of genes involved in such processes and their adaptation and re-functionalization during evolution it is difficult to define developmental gene sets in e.g. plants and animals. The social amoebae can serve as a relative simple model for development. A further advantage is that for over 600 mya the developmental cycle remained stable with only a few changes. This enables the study of conserved, and therefore possibly important, functions, of this developmental program. We were able to define a small number of potentially developmentally relevant genes, of which a large number so far escaped functional studies. Our study thus will help the research community interested in development to get further insights in the evolution and maintenance of such programs.

## Additional files

Additional file 1: Table S1. Gene set: The genes identified and additional information on orthology, detection method, and expression peak. (XLSX 97 kb)
Additional file 2: Table S3. Oligonucleotides primer sequences for knock out studies. (DOC 56 kb)
Additional file 3: Supplementary Figures 1-6. (DOCX 3657 kb)
Additional file 4: Table S2. Upregulated mutants upregulated mutant list and ko phenotype description. (XLSX 22 kb)
