# Supplementary material for: A set of genes conserved in sequence and expression traces back the establishment of multicellularity in social amoebae
Source: BMC Genomics. 2016 Nov 4;17:871. doi: 10.1186/s12864-016-3223-z (PMC5097433; doi:10.1186/s12864-016-3223-z)
Supplement: Additional file 2: Table S3. — Oligonucleotides primer sequences for knock out studies. (DOC 56 kb) [file 12864_2016_3223_MOESM2_ESM.doc]

Supplemental table 3. Oligonucleotide primers for knock out studies used in this work

| Gene | Name | Restriction site | DNA sequence 5’-3’ |
| --- | --- | --- | --- |
| DDB_G0275521 | 521-KO1-5' | KpnI | GGGGTACCCAATATATAATAATAGAGAAA |
|  | 521-KO1-3' | HindIII | CCCAAGCTTCCAAAACAAAAGAAAATTTA |
|  | 521-KO2-5' | BamHI | CGGGATCCGGATTACCAGATAGGCAA |
|  | 521-KO2-3' | NotI | GCGGCCGCGGTGAACATCATCAGTAT |
|  | 521-neg5' |  | GGGTAAATATTAATTGATTTTTTT |
|  | 521-neg3' |  | CCATAAATGAAAAATAACAACATT |
|  | 521-pos5' |  | CCAATGACTATCATTGGAATTC |
|  | 521-pos3' |  | GGTTTTGATGAATAATTATAATGAT |
| pLPBLP | 5'Lox |  | CACCGTGGTTAATTAATTAACCCGGGAA |
|  | 3'Lox |  | GCCGCATGGTTAATTCCTGCAG |
| DDB_G0287037 | 037-KO1-5' | KpnI | GGGGTACCCCATCAACATCAACAACTC |
|  | 037-KO1-3' | HindIII | CCCAAGCTTCTGGTGCTGAAAAAACTTT |
|  | 037-KO2-5' | BamHI | CGGGATCCGGGTGCGGTTGGGAA |
|  | 037-KO2-3' | NotI | GCGGCCGCGGATATTTCAAAAACAGCA |
|  | 037-neg5' |  | GCACCAGTTTTAGGTGAAGA |
|  | 037-neg3' |  | CCCAAGTAATGGATGTTTTGG |
|  | 037-pos5' |  | GGTAATTTGAAACAACAATTTCAA |
|  | 037-pos3' |  | CCATTAAAAAGAATTGGTAATACA |
| DDB_G0269826 | 826-KO1-5' | KpnI | GGGGTACCGGGAATAAAAATAACCCTT |
|  | 826-KO1-3' | HindIII | CCCAAGCTTCCTAGTTGTTCATTTAAATAA |
|  | 826-KO2-5' | BamHI | CGGGATCCGCAATCAAATCAATATTTACA |
|  | 826-KO2-3' | NotI | GCGGCCGCCCAATTATTATCAACTATTT |
|  | 826-neg5' |  | GAACAACTAGGAATTAATAGTAA |
|  | 826-neg3' |  | GCTTTAAAAGTATTTCAAATACTT |
|  | 826-pos5' |  | GTAAATTTCCATTTTGCCGGAA |
|  | 826-pos3' |  | CCATTTGGTGTTTTATCTCTTTT |
| DDB_G0288963 | 963-KO1-5' | KpnI | GGGGTACCCGATTAGTGTTGGTATTTTCAA |
|  | 963-KO1-3' | HindIII | CCCAAGCTTCCAACTGAATTGATTGTATAAA |
|  | 963-KO2-5' | BamHI | CGGGATCCGGTAAATCATCATTGGGTA |
|  | 963-KO2-3' | NotI | GCGGCCGCCCATTTGAGGGGTCAGTT |
|  | 963-neg5' |  | GGTCAACCATTGACAAGGGATT |
|  | 963-neg3' |  | CCAATTGAATATAATTTTGATTGATT |
|  | 963-pos3' |  | CCAAAACTTGAAACTGTTACATTATA |
|  | 963-pos5' |  | GGTAGTATTTGAATATGAGTGTG |
| DDB_G0272550 | 550-KO1-5' | NotI | GCGGCCGCGGAAGGTATTGATAATGAAGTT |
|  | 550-KO1-3' | BamHI | CGGATCCTTGTGGTTTACGTTT |
|  | 550-KO2-5' | HindIII | CCCAAGCTTGGTATGTTTTTGTCTGTGATT |
|  | 550-KO2-3' | KpnI | GGGTACCCCATAATCAATACTATTACCCAAA |
|  | 550-neg5' |  | CCGTTTTATCAACTGCATTCT |
|  | 550-neg3' |  | CCGGTACCCATTCTACGAA |
|  | 550-pos5' |  | GGCGATAGAAGAAGAATCATCAAA |
|  | 550-pos3' |  | CCACAATAATTAAATACTAGAATTATTT |
