# Supplementary material for: A set of genes conserved in sequence and expression traces back the establishment of multicellularity in social amoebae
Source: BMC Genomics. 2016 Nov 4;17:871. doi: 10.1186/s12864-016-3223-z (PMC5097433; doi:10.1186/s12864-016-3223-z)
Supplement: Additional file 3: — Supplementary Figures 1-6. (DOCX 3657 kb) [file 12864_2016_3223_MOESM3_ESM.docx]

Figure S1: Principal component analysis of DD samples at different time points. The analysis was done using cummeRbund [[1](#_ENREF_1)] based on the counts generated with HTseqcount [[2](#_ENREF_2)]. Each data point represents a gene and the red arrows indicate the directionality of all genes for a given sample.


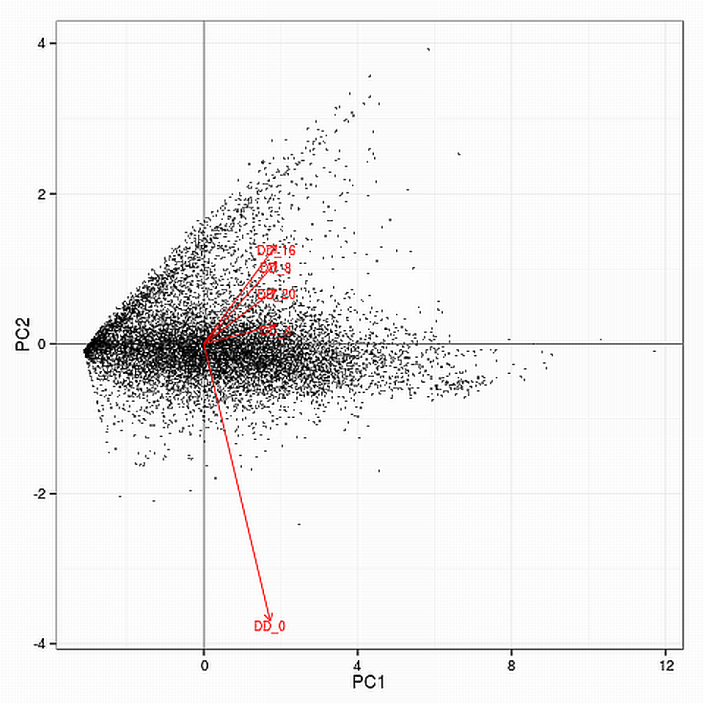


Figure S2: Term enrichments in genes defined with method A (>=3 x differential expression) as defined in the text. GO terms were analysed using the generic GOterm finder [[3](#_ENREF_3)] and the resulting enriched lists visualized with Revigo [[4](#_ENREF_4)].


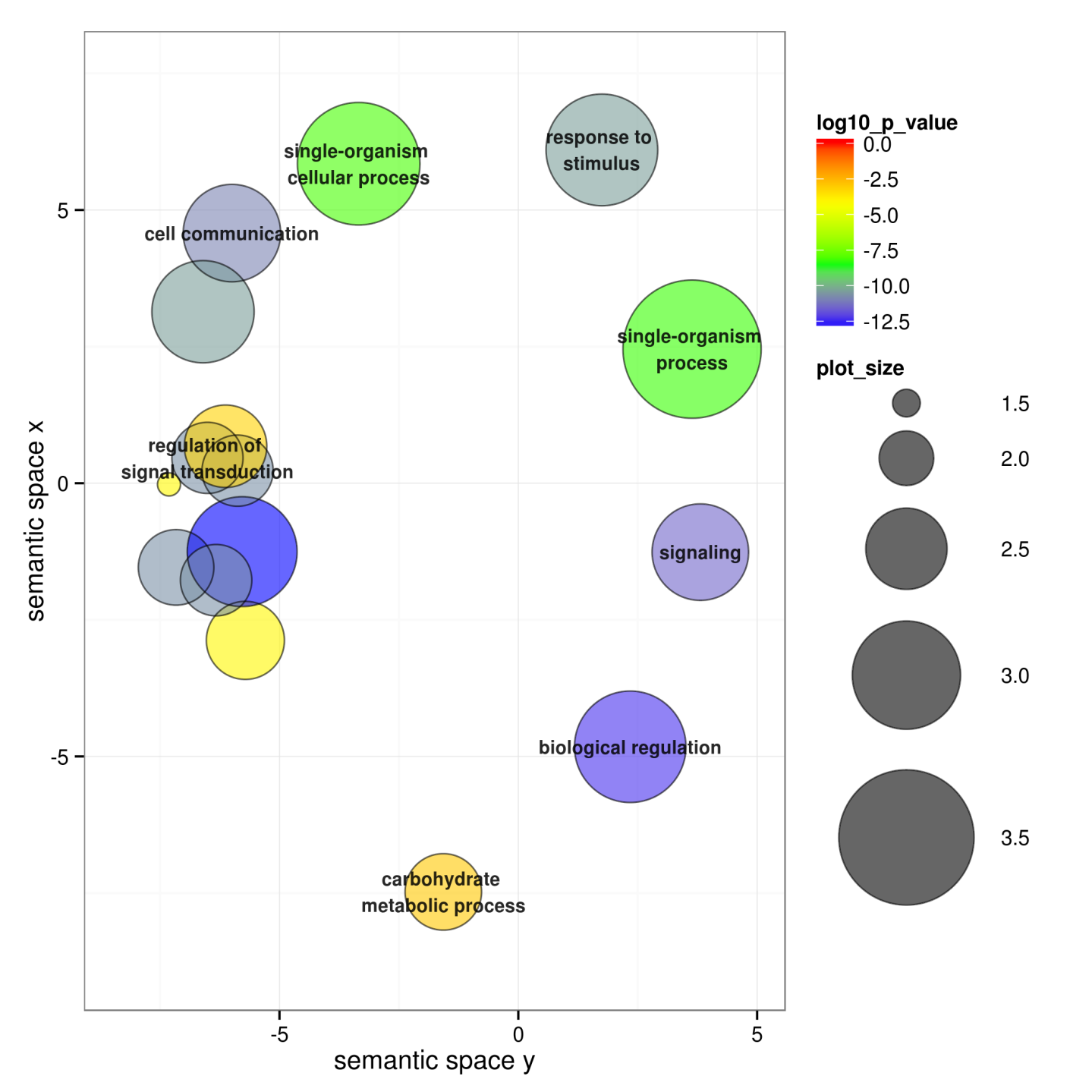


Figure S3: Conservation is correlated to peak expression in the defined developmental set. Genes were grouped according to their peak expression during the developmental cycle (t_1 = 4 h; t_2 = 8 h; t_3 = 16 h; t_4 = 20 h). Conservation was measured as identity between DD and DF proteins. A Wilcoxon-Mann-Whitney-Test implemented in R was used to analyse the statistical significance of differences between groups. The significance (bars with asterisks) between groups t_1 and t_3 was 0.00309 and between t_1 and t_4 0.00089. All other pairs showed no significant difference with this test. Outliers within groups are depicted as black dots.


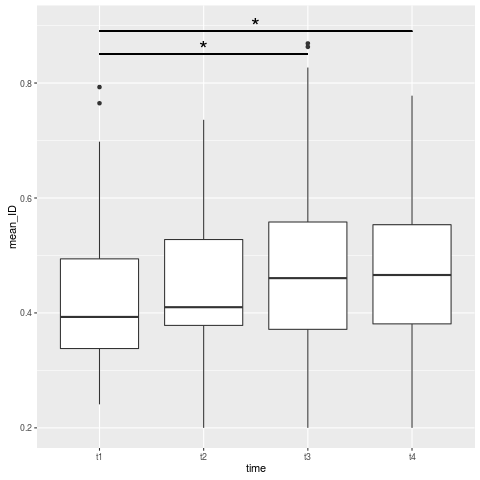
.

Figure S4: Knock-out constructs and PCR diagnosis

A. Knock-out (KO) constructs consist of the floxed A15::Bsr cassette flanked by two fragments, amplified using primers complementary to a 5’ and a 3’ region of ~1 kb of the target gene. After homologous recombination a section of the target gene is replaced by the floxed A15::Bsr cassette. Knock-outs were diagnosed by the absence of a PCR product amplified by primers inside the deleted region (negative control) and the presence of PCR products amplified by primers inside the selection cassette and outside the 3’ or 5’ KO fragments (5’ and 3’positive controls).

B. PCR products using primers for amplification of the 5’ positive control fragment from genomic DNAs of wild-type AX2 cells (WT), and some KO and random integrant (RI) clones for four target genes.


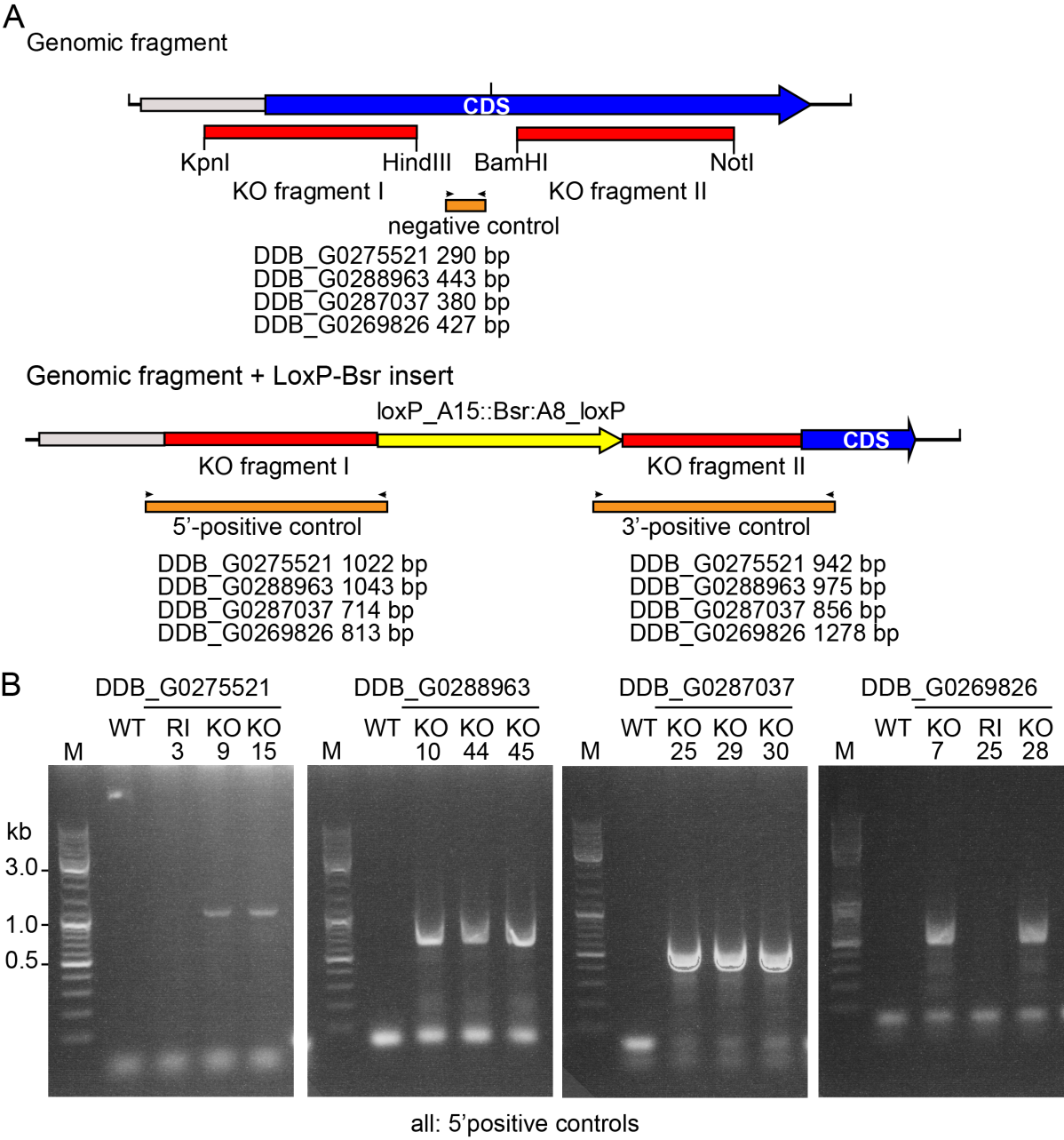


Figure S5: Development and terminal cell type differentiation of wild type *D.discoideum* AX2 cells and knockout (KO) clones. AX2 is the axenic version of the original NC4 isolated strain. Details of strain histories can be obtained from the dictybase.org web page.

A. Cells were grown in rich medium, washed and then developed on non-nutrient agar. Photographs were taken at 2 hour intervals. Only images taken at 8 h and 24 h are shown. No major differences in developmental progression between AX2 wild type cells and the different knockouts were detected. All knockouts formed aggregation streams and morphologically normal fruiting bodies.

B. Fruiting bodies of wildtype and KO clones were transferred to a droplet of 0.002% Calcofluor White (an agent interacting with cellulose) on a slide glass and photographed under UV illumination. All knockout clones showed strong cellulose staining of elliptical spores and normal architecture of cellulose encased stalk cells enclosed by the cellulose stalk tube.


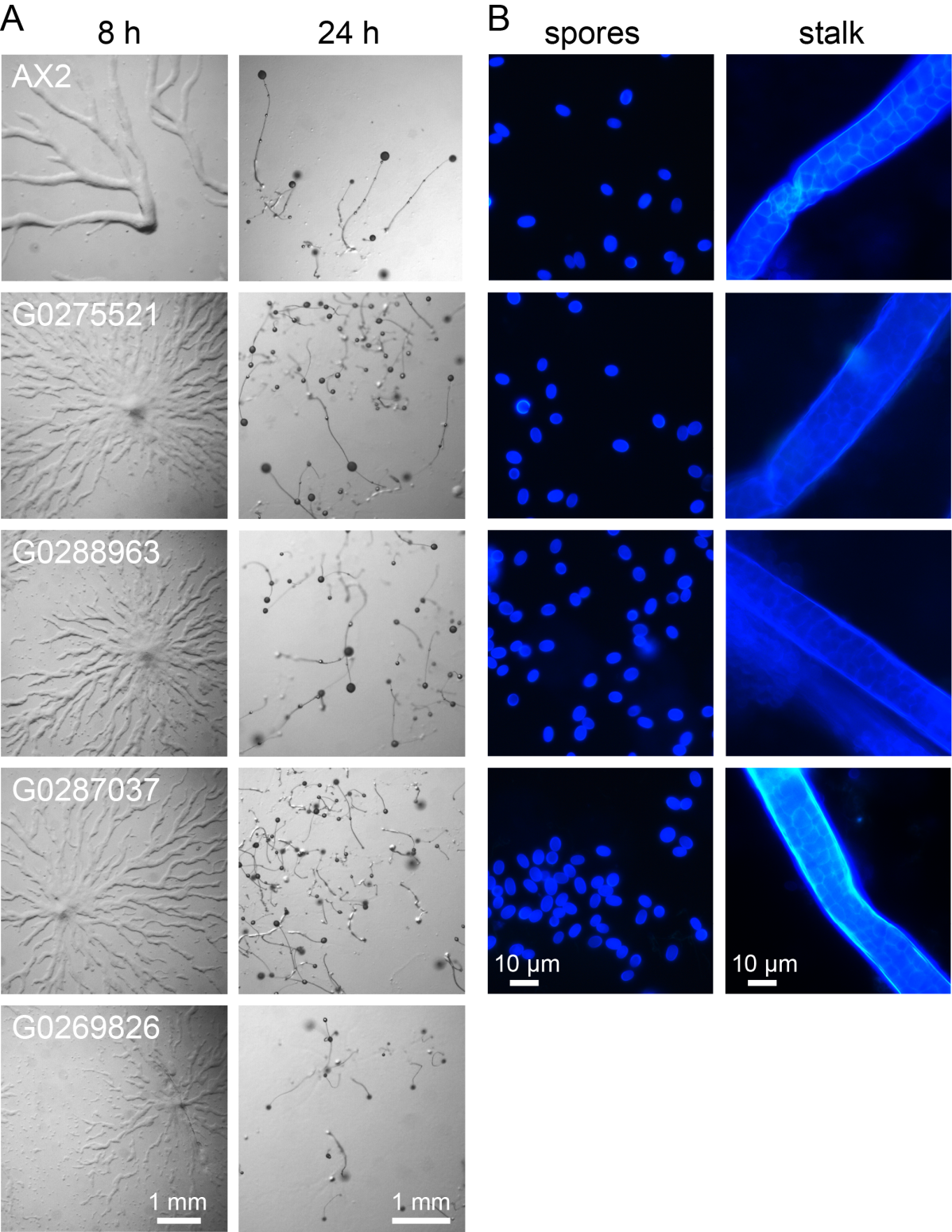


Figure S6: Spore associated properties

A. *Spore viability*. AX2 Wild-type, knock-out (KO) and random integrant (RI) spores, harvested from mature fruiting bodies were treated for 10 min with 0.1% TritonX-100 to lyse amoeboid cells, counted and plated at 100 spores per plate on SM agar with *Klebsiella aerogenes*. Emerging colonies were counted after 3 days. Means and SD of two experiments are shown. Compared to the wild type, most KO and RI clones formed the same or a higher number of colonies, which indicates that there are no defects in spore germination in the knockouts. The higher number of colonies may be due to errors in spore counting. One RI clone seemed to yield significantly less clones. Gene DDB_G0269826 was the last gene to be knocked-out after several efforts and was not included in these initial experiments.

B. *Sporulation efficiency*. 60,000 cells were developed into fruiting bodies on a 1 cm^2^ nitrocellulose filter, supported by non-nutrient agar. The filter plus fruiting bodies were shaken in 1 ml 0.1% TritonX-100 for 10 minute, spores were collected by centrifugation and counted. Some individual KO or RI clones performed worse than the wild type in this experiment, but this was not true for all clones of the same transformation. We therefore concluded that overall, there is no difference in sporulation efficiency.

C. *Competition with wild-type over 5 generations*. Mature spores of KO and RI clones were mixed at a 1:1 ratio with AX2 wild type spores and developed on *K. aerogenes* lawns over five generations of fruiting body formation and re-inoculation of 0.1% TritonX-100 treated spores. Spores were then harvested from the chimeric fruiting bodies, treated with 0.1% TritonX-100 and plated clonally. From each chimeric mixture, cells from 96 clones were transferred into 100 µl HL5 supplemented with 10 µg/ml blasticidin and wells with surviving growing cells were scored after 4 days. Means and SD of two 96 well plates for each mixture are shown. The control is a plate seeded from clones of a blasticidin resistant mutant clone that was not mixed with wild-type cells.


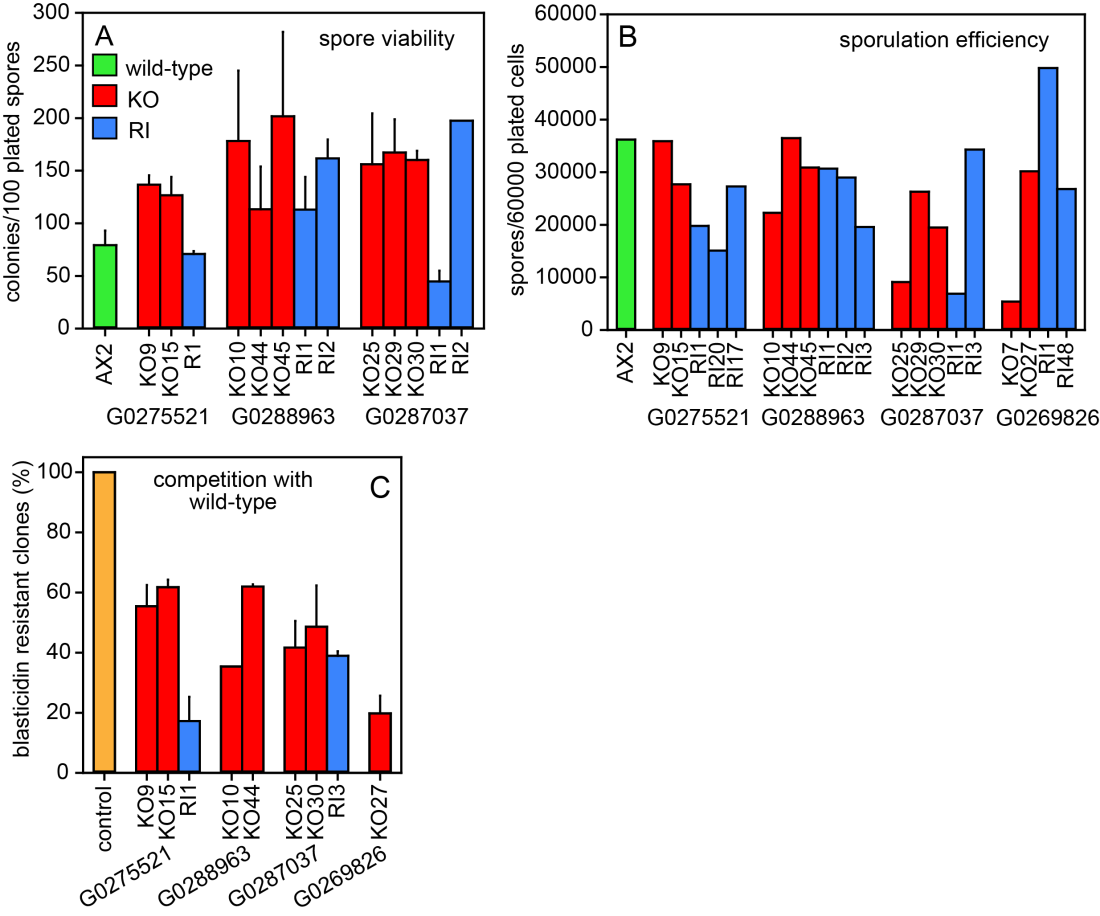


1. Trapnell C, Roberts A, Goff L, Pertea G, Kim D, et al. (2012) Differential gene and transcript expression analysis of RNA-seq experiments with TopHat and Cufflinks. Nat Protoc 7: 562-578.

2. Anders S, Pyl PT, Huber W (2014) HTSeq- A Python framework to work with high-throughput sequencing data. bioRxiv preprint.

3. Boyle EI, Weng S, Gollub J, Jin H, Botstein D, et al. (2004) GO::TermFinder--open source software for accessing Gene Ontology information and finding significantly enriched Gene Ontology terms associated with a list of genes. Bioinformatics 20: 3710-3715.

4. Supek F, Bosnjak M, Skunca N, Smuc T (2011) REVIGO summarizes and visualizes long lists of gene ontology terms. PLoS One 6: e21800.
